# Three-years of dalbavancin use at a UK tertiary referral hospital serving a population with high numbers of people who inject drugs

**DOI:** 10.1093/jacamr/dlae066

**Published:** 2024-05-06

**Authors:** Carolin Bresges, Kristina Bresges, Claudette Hewitt, Sunil Sharma, Bethany Davies

**Affiliations:** Brighton and Sussex Medical School Department of Global Health and Infection, University of Sussex, 94 N–S Road, Falmer, Brighton BN1 9PX, UK; The Royal Sussex County Hospital, University Hospitals Sussex NHS Foundation Trust, Eastern Road, Brighton BN2 5BE, UK; Department of Anaesthetics, King’s College Hospital NHS Foundation Trust, Denmark Hill, London SE5 9RS, UK; The Royal Sussex County Hospital, University Hospitals Sussex NHS Foundation Trust, Eastern Road, Brighton BN2 5BE, UK; The Royal Sussex County Hospital, University Hospitals Sussex NHS Foundation Trust, Eastern Road, Brighton BN2 5BE, UK; Brighton and Sussex Medical School Department of Global Health and Infection, University of Sussex, 94 N–S Road, Falmer, Brighton BN1 9PX, UK; The Royal Sussex County Hospital, University Hospitals Sussex NHS Foundation Trust, Eastern Road, Brighton BN2 5BE, UK

## Abstract

**Background:**

Dalbavancin’s unique properties have led to an increase in its off-licence use in complex infection and in vulnerable populations including people who inject drugs (PWID), but data remain limited. In this retrospective cohort study, we describe the characteristics, treatment rationale and outcomes for all adult inpatients treated with dalbavancin at a UK tertiary hospital.

**Results:**

Fifty-eight inpatients were treated with dalbavancin between 1 January 2018 and 1 January 2021, 98.3% for off-licence diagnoses. Acute bacterial skin and skin structure infection, infective endocarditis and endovascular infections were each diagnosed in 22.4% of patients. Bone and joint infections were diagnosed in 18.9%, discitis in 12.1% and central line-associated bloodstream infections in 5.2%. Sixty-nine percent of patients were bacteraemic; 52.5% *Staphylococcus aureus*, 5.0% MRSA. Two mild adverse reactions were attributed to dalbavancin. Treatment was successful in 43 (75.4%) patients, and failed in seven (12.3%). Seven (12.3%) were lost to follow-up.

Thirty-five patients (60.3%) were PWID, with low median age (41.0 years) and Charlson Comorbidity scores (0). Self-discharge was taken by 17.1% of PWID, and 20.6% were lost to follow-up. At 90 days, three (8.6%) PWID were deceased.

**Conclusions:**

In this first UK cohort, dalbavancin was used off licence and in persons facing barriers to conventional therapies. Where data is available, it was safe and effective. Dalbavancin appears a potentially valuable tool in improving outcomes for PWID.

## Background

Gram-positive bacteria are a major cause of infection worldwide, and associated with high antimicrobial resistance, morbidity, mortality and health expenditure.^1–3^ Dalbavancin is a semisynthetic lipoglycopeptide with activity against staphylococci, streptococci and vancomycin-susceptible enterococci. The lipophilic side chains confer the long terminal half-life of 8.5 days.^4^ Like Europe and the USA, in the UK dalbavancin is licenced for the treatment of acute bacterial skin and skin structure infection (ABSSSI) in non-pregnant adults caused by susceptible Gram-positive bacteria including MRSA. Studies have demonstrated it is well tolerated and safe,^5^ and it can reduce the length of inpatient stays and associated costs.^6^ Owing to its unique properties, the off-licence use of dalbavancin in complex infections has increased, particularly in those facing barriers to conventional management. However, data remain limited.^7^

In 2018, when our study began, an estimated 1.3 million adults had high-risk opioid use in the European Union, with the highest prevalence in the UK at 0.82%.^8^ People who inject drugs (PWID) are disproportionately affected by bacterial infections, most commonly due to Gram-positive organisms. They face multiple barriers to healthcare, including access to long-term intravenous lines, issues with adherence, high numbers of self-discharges and losses to follow-up (LTF).^9–11^

This first published UK cohort describes the characteristics, clinical management and outcomes for adult inpatients treated with dalbavancin over 3 years at a tertiary hospital serving a population including high numbers of PWID.

## Methods

### Study design and procedures

We conducted a retrospective case-note review at the Royal Sussex County Hospital (RSCH), a tertiary referral hospital in Brighton, UK, serving approximately 1.8 million people. The estimated prevalence of intravenous drug use is 2% of people aged 15–44 years.^12^ All dalbavancin treatments are at the discretion of an infection specialist. There are no dedicated infection services for PWID.

Pharmacy records identified sequential inpatients for whom dalbavancin was ordered between 1 January 2018 and 1 January 2021. Patients were included if they were aged 18 years or above, admitted to hospital during the study period, and received dalbavancin for an acute infection episode. Patients receiving multiple courses of dalbavancin were included for each distinct infection. Absence of necessary data excluded patients.

Data were collected on patient characteristics, inpatient investigations, clinical diagnoses, management, dalbavancin treatment rationale and goals. Inpatient and infection episodes outcomes were extracted, and all-cause mortality recorded 90 days after first dalbavancin dose. Anonymized data were managed and analysed in Microsoft Excel v.16.66.

### Ethical considerations

Non-identifiable data were collected to maximize the anonymity of the cohort. As a retrospective review, patient care was not affected. In accordance with the University Hospitals Sussex Trust Research and Development department, and with the NHS Health Research Authority, this study did not necessitate ethical approval.

### Definitions and statistical methods

Patient sex was the biological sex recorded in clinical notes. A Charlson Comorbidity Index Score was calculated to reflect prognostic comorbidity as described elsewhere.^13^ ‘Insecure accommodation’ was recorded for those who were homeless, sofa surfing or staying in hostels. All inpatient diagnoses were recorded as defined by the treating physicians. ‘Endovascular infection’ includes permanent-pacemaker infection. Microbiological diagnosis was when a pathogen considered causative of the infection episode treated with dalbavancin was identified. Surgical intervention includes any procedure relating to the infection episode. Antimicrobial therapy other than dalbavancin was quantified by ‘days of therapy’ (DOT) as defined elsewhere.^14^ Categories for dalbavancin rationale include ‘healthcare worker concerns around long-term intravenous access’, ‘patient conduct on ward’, ‘non-adherence to conventional therapy’, ‘antimicrobial contraindications’, ‘palliative approach’ and ‘barriers to conventional therapy’. The last group was heterogenous, including drug interactions, unstable housing, accessibility and cognitive barriers.

The outcome of treatment with dalbavancin was assessed by the data-collecting study physicians (C.B., K.B., C.H.) at 90 days after first dose. Where possible, patients’ hospital, Summary Care Records and, where deceased, death certificates were accessed. Treatment was considered a ‘success’ if there were no additional treatments nor admissions relating to the infection episode treated with dalbavancin. Where follow-up was planned, contact with a healthcare professional without documented concern ongoing. Treatment was considered a ‘failure’ if the criteria for success where not met. In the case of death, failure was recorded if the causes of death related to infection treated with dalbavancin. ‘Lost to follow-up’ was applied where follow-up was planned within the 90-day period and there was no evidence of assessment by a healthcare professional. Where no data were accessible after discharge, outcomes were considered ‘unknown’. These definitions were independent of patient compliance with planned therapies. Where the treatment outcome was in doubt, the data-collecting physicians conferred and, if necessary, referred to a third senior study physician (B.D. or S.S.). Adverse reactions to dalbavancin were as per the Medicines and Healthcare products Regulatory Agency definition and identified by the study physicians collecting data.^15^

Analyses were descriptive, providing frequencies and proportions for categorical variables, and medians with their IQR for continuous variables with skewed distributions. Data disaggregated by history of intravenous drug use are presented. Persons with current or history of illicit intravenous drug use within the previous 12 months were included in the PWID group due to the shared risk of complicated infections with metastatic seeding due to a higher likelihood of abnormal anatomy, as well as comparable rationale for dalbavancin use.

## Results

Baseline patient characteristics are presented in Table 1, and diagnoses, clinical management and outcomes are presented in Table 2. Table 3 presents the data for patients for whom therapy with dalbavancin failed.

**Table 1.** The baseline characteristics of adult inpatients treated with dalbavancin over a 3-year period at a large teaching hospital in Brighton

	Whole cohort	Non-PWID group	PWID group
Number of patients	58	23	35
Age at admission, years, median (IQR)	46.0 (38.5–59.8)	72.0 (53.0–79.5)	41.0 (32.5–45.0)
Male sex (%)	37 (63.8)	15 (65.2)	22 (62.9)
Accommodation on admission (%)	*n* = 55		*n* = 32
Own home	32 (58.2)	19 (82.6)	13 (40.6)
Insecure housing	19 (34.5)	1 (4.3)	18 (56.3)
Health facility	4 (7.3)	3 (13.0)	1 (3.1)
Length of inpatient stay, days, median (IQR)	15.5 (9.3–27.0)	26.0 (10.5–49.5)	14.0 (9.5–19.0)
Number of days from first dalbavancin dose to day of discharge, median (IQR)	1.0 (0–6.8)	4.0 (0–11.0)	0 (0–3.0)
Admission outcome (%)			
Discharged	50 (86.2)	21 (91.3)	29 (82.9)
Self-discharged	6 (10.3)	0	6 (17.1)
Died	2 (3.4)	2 (8.7)	0
Comorbidities (%)			
History of VTE	20 (34.5)	2 (8.7)	18 (51.4)
HCV	19 (32.8)	0	19 (54.3)
Diabetes	12 (20.7)	10 (41.6)	2 (5.7)
Depression	10 (17.2)	1 (4.3)	9 (25.7)
Malignancy	7 (12.1)	7 (30.4)	0
Dementia	3 (5.2)	3 (13.0)	0
HIV	0	0	0
HBV	0	0	0
Charlson Comorbidity Index Score, median (IQR)	1.0 (0–4.0)	5.0 (2.5–7.5)	0 (0–0.5)
Admission bloods, median (IQR)			
Hb (g/dL)	11.1 (9.9–12.3)	11.0 (9.1–12.0)	11.2 (10.1–12.3)
WCC (×10^9^/L)	10.2 (7.6–13.3)	11.8 (8.3–17.3)	9.4 (7.5–12.9)
Neutrophils (×10^9^/L)	8.0 (6.1–9.8)	9.1 (6.3–13.6)	7.4 (5.8–9.5)
CRP (mg/L)	105.5 (49.3–188.5)	68.0 (34.0–114.0)	125.0 (64.5–229.0)
Creatinine (mmol/L)	78.0 (63.0–106.0)	112 (91.0–138.5)	70.0 (58.5–79.0)
Albumin (g/L)	38.0 (32.0–42.0) (*n* = 53)	37.0 (29.0–43.0) (*n* = 21)	38.5 (32.8–41.3) (*n* = 32)

Data are presented for the whole cohort, for PWID and for those without a history of illicit intravenous drug use (non-PWID).

For the whole cohort *n* = 58 unless otherwise stated; for the non-PWID group *n* = 23 unless otherwise stated; for the PWID group *n* = 35 unless otherwise stated.

VTE, venous thromboembolism; HBV, Hepatitis B virus; Hb, haemoglobin (reference range for adult females: 11.5–16.5 g/dL; reference range for adult males: 13.5–18.0 g/dL); WCC, white cell count (reference range: 4.0–10.0 × 10^9^/L; neutrophil reference range: 2.0–7.0 × 10^9^/L).

**Table 2.** The diagnoses, clinical management and outcomes of adult inpatients treated with dalbavancin over a 3-year period at a large teaching hospital in Brighton

	Whole cohort	Non-PWID group	PWID group
Diagnostic indications for dalbavancin (%)			
Bacteraemia	40 (69.0)	17 (73.9)	23 (65.7)
ABSSSI	13 (22.4)	4 (17.4)	9 (25.7)
IE [prosthetic valve]	13 (22.4) [5 (8.6)]	8 (34.8) [5 (21.7)]	5 (14.3) [0]
Endovascular infection	13 (22.4)	2 (8.7)	11 (31.4)
Native bone and joint infection	9 (15.5)	2 (8.7)	7 (20.0)
Discitis	7 (12.1)	3 (13.0)	4 (11.4)
CLABSI	3 (5.2)	3 (13.0)	0
Prosthetic bone and joint infection	2 (3.4)	2 (8.7)	0
Diagnoses associated with bacteraemias (%)	*n* = 40	*n* = 17	*n* = 23
IE [prosthetic valve]	11 (27.5) [5 (12.5)]	7 (41.2) [5 (29.4)]	4 (17.4) [0]
ABSSSI	7 (17.5)	1 (5.9)	6 (26.1)
Endovascular infection	7 (17.5)	2 (11.8)	5 (21.7)
Unknown origin	5 (12.5)	2 (11.8)	3 (13.0)
Native bone and joint infection	5 (12.5)	1 (5.9)	4 (17.4)
Discitis	5 (12.5)	2 (11.8)	3 (13.0)
CLABSI	3 (7.5)	3 (17.6)	0
Prosthetic bone and joint infection	0	0	0
Organisms causing bacteraemia (%)	*n* = 40	*n* = 17	*n* = 23
MSSA	21 (52.5)	7 (41.2)	14 (60.9)
*Streptococcus pyogenes*	4 (10.0)	0	4 (17.4)
*Enterococcus faecalis*	4 (10.0)	4 (23.5)	0
*Staphylococcus epidermidis*	3 (7.5)	3 (17.6)	0
*Streptococcus dysgalactiae*	2 (5.0)	0	2 (8.7)
MRSA	2 (5.0)	1 (5.9)	1 (4.3)
Mixed bacteraemia	2 (5.0)	0	2 (8.7)
*Streptococcus mitis* group	1 (2.5)	1 (5.9)	0
Known MRSA colonized (%)	5 (8.6)	2 (8.7)	3 (8.6)
Microbiological diagnosis (%)	46 (79.3)	19 (82.6)	27 (77.1)
Surgical intervention as part of management (%)	18 (31.0)	7 (30.4)	11 (31.4)
Antimicrobials during admission before dalbavancin			
Patient receiving ≥1 ABX before dalbavancin (%)	58 (100)	24 (100)	35 (100)
Number of ABX before dalbavancin, median (IQR)	3.0 (2.0–4.0)	3.0 (2.0–5.0)	2.0 (2.0–4.0)
Duration of ABX before dalbavancin, DOT, median (IQR)	18.0 (7.0–28.0) (*n* = 56)	19.0 (8.0–51.8) (*n* = 22)	17.5 (6.0–22.8) (*n* = 34)
Rationale for dalbavancin (%)	*n* = 56	*n* = 21	*n* = 35
Barriers to conventional therapy	26 (46.4)	9 (42.9)	17 (48.6)
Healthcare worker concerns around long-term IV access	25 (44.6)	4 (19.0)	21 (60.0)
Patient conduct on ward	14 (25.0)	1 (4.8)	13 (37.1)
Non-adherence to conventional therapy	13 (23.2)	2 (9.5)	11 (31.4)
Antimicrobial contraindications	6 (10.7)	5 (23.8)	1 (2.9)
Palliative approach	4 (7.1)	4 (19.0)	0
Other	2 (3.6)	1 (4.8)	1 (2.9)
Dalbavancin therapy			
Cumulative dalbavancin dose (mg), median (IQR)	1500 (1500–3000) (*n* = 56)	2000 (1500–3000)	1500 (1500–3000) (*n* = 33)
Number of dalbavancin doses per patient, median (IQR)	1.0 (1.0–2.0)	2.0 (1.0–2.0)	1.0 (1.0–2.0)
Number of patients who missed ≥1 planned dose (%)	8 (13.8)	0	8 (22.9)
Number of patients treated with concurrent antimicrobials (%)	14 (24.1)	6 (26.1)	8 (22.9)
Treatment outcomes (%)	*n* = 57	*n* = 23	*n* = 34
Success	43 (75.4)	19 (82.6)	24 (70.6)
Failure	7 (12.3)	4 (17.4)	3 (8.8)
Lost to follow-up	7 (12.3)	0	7 (20.6)
Treatment adverse reactions (%)	2 (3.4)	1 (4.3)	1 (2.9)
Patients deceased within 90 days of first dalbavancin dose (%)	6 (10.3)	3 (13.0)	3 (8.6)

Data are presented for the whole cohort, for PWID and for those without a history of illicit intravenous drug use (non-PWID).

For the whole cohort *n* = 58 unless otherwise stated; for the PWID group *n* = 23 unless otherwise stated; for the non-PWID group *n* = 35 unless otherwise stated.

CLABSI, central line-associated bloodstream infection; ABX, antibiotics.

**Table 3.** The baseline characteristics diagnoses, clinical management and outcomes of adult inpatients whose treatment with dalbavancin ‘failed’ over a 3-year period at a large teaching hospital in Brighton

	Whole cohort	Non-PWID group	PWID group
Number of patients	7	4	3
Age at admission, years, median (IQR)	46 (35.0–64.0)	64 (51.3–77.5)	32 (30.5–35.0)
Male sex (%)	4 (57.1)	2 (50)	2 (66.7)
Accommodation on admission (%)			
Own home	3 (42.9)	3 (75)	0
Insecure housing	3 (42.9)	0	3 (100)
Health facility	1 (14.3)	1 (25)	0
Length of inpatient stay, days, median (IQR)	12 (5.0–20.5)	10 (5.0–24.3)	12.0 (8.0–19.5)
Admission outcome (%)			
Discharged	5 (71.4)	2 (50)	3 (100)
Self-discharged	0	0	0
Died	2 (28.6)	2 (50)	0
Charlson Comorbidity Index Score, median (IQR)	1.0 (0–5.0)	5.0 (4.0–5.3)	0 (0–0)
Diagnostic indications for dalbavancin (%)			
Bacteraemia	3 (42.9)	1 (25)	2 (66.7)
ABSSSI	2 (28.6)	2 (50)	0
IE [prosthetic valve]	2 (28.6) [1 (14.3)]	2 (50) [1 (25.0)]	0
Endovascular infection	1 (14.3)	0	1 (33.3)
Native bone and joint infection	3 (42.9)	1 (25)	2 (66.7)
Discitis	0	0	0
CLABSI	0	0	0
Prosthetic bone and joint infection	0	0	0
Organisms causing bacteraemia (%)	*n* = 3	*n* = 1	*n* = 2
MSSA	2 (66.7)	0	2 (100)
*Streptococcus pyogenes*	0	0	0
*Enterococcus faecalis*	0	0	0
*Staphylococcus epidermidis*	0	0	0
*Streptococcus dysgalactiae*	0	0	0
MRSA	0	0	0
Mixed bacteraemia	0	0	0
*Streptococcus mitis* group	1 (33.3)	1 (100)	0
Microbiological diagnosis (%)	3 (42.9)	1 (25)	2 (66.7)
Surgical intervention as part of management (%)	3 (42.9)	1 (25)	2 (66.7)
Antimicrobials during admission before dalbavancin			
Patient receiving ≥1 ABX before dalbavancin (%)	7 (100)	4 (100)	3 (100)
Number of ABX before dalbavancin, median (IQR)	2.0 (2.0–4.5)	3.0 (2.0–4.3)	2.0 (1.5–3.5)
Duration of ABX before dalbavancin, DOT, median (IQR)	9.0 (5.0–14.0)	7.0 (4.3–19.5)	10.0 (7.5–14.0)
Rationale for dalbavancin (%)			
Barriers to conventional therapy	3 (42.9)	1 (25)	2 (66.7)
Healthcare worker concerns around long-term IV access	2 (28.6)	1 (25)	1 (33.3)
Patient conduct on ward	1 (14.3)	0	1 (33.3)
Non-adherence to conventional therapy	2 (28.6)	0	2 (66.7)
Antimicrobial contraindications	0	0	0
Palliative approach	2 (28.6)	2 (50)	0
Other	0	0	0
Dalbavancin therapy			
Cumulative dalbavancin dose (mg), median (IQR)	1500 (1500–2625) (N = 6)	1500 (1375–1875)	2250 (1875–2625) (N = 2)
Number of dalbavancin doses per patient, median (IQR)	1.0 (1.0–1.5)	1.0 (1.0–1.25)	1.0 (1.0–1.5)
Number of patients who missed ≥1 planned dose (%)	1 (14.3)	0	1 (33.3)
Number of patients treated with concurrent antimicrobials (%)	2 (28.6)	1 (25)	1 (33.3)
Treatment adverse reactions (%)	1 (14.3)	0	1 (33.3)
Patients deceased within 90 days of first dalbavancin dose (%)	2 (28.6)	2 (50)	0

Data are presented for the whole cohort, for PWID and for those without a history of illicit intravenous drug use (non-PWID).

For the whole cohort, *n* = 7 unless otherwise stated; for the non-PWID group *n* = 4 unless otherwise stated; for the PWID group *n* = 3 unless otherwise stated.

CLABSI, central line-associated bloodstream infection; ABX, antibiotics.

### Baseline patient characteristics

**Figure 1.**
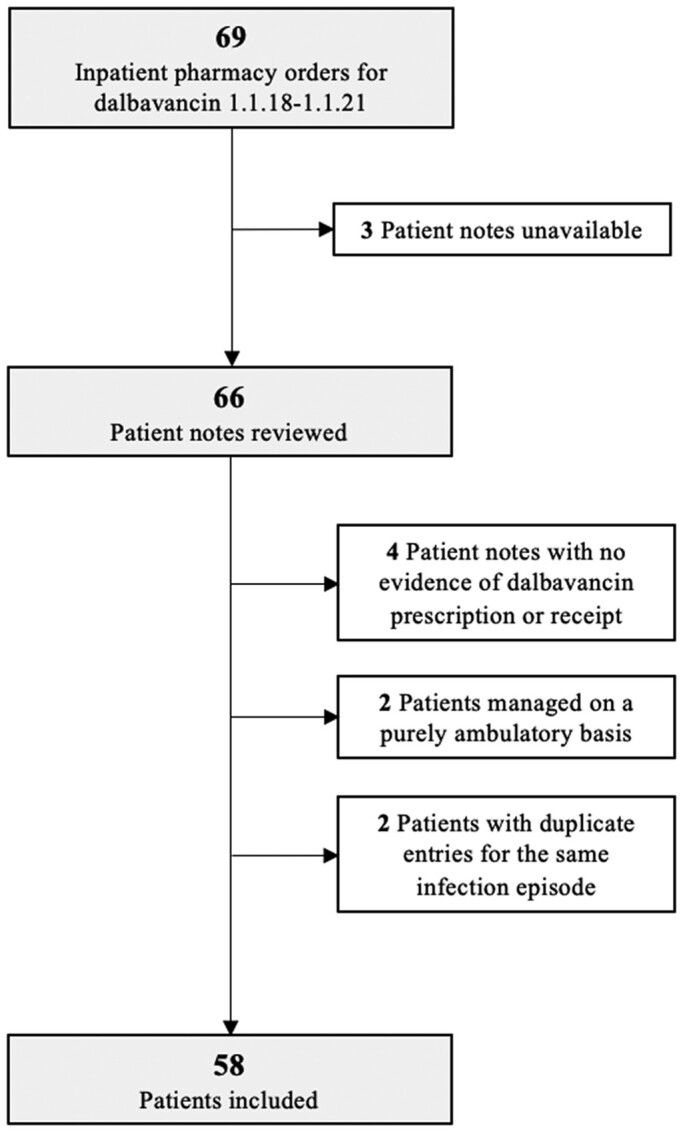
Patient flow diagram.

Of the 69 pharmacy orders for dalbavancin, 58 (84.1%) met our inclusion criteria (Figure 1). The median age was 46.0 years (IQR 38.5–59.8) and 63.8% (37/58) were male. At the time of admission, 34.5% (19/58) had insecure accommodation. Thirty-five (60.3%) had a history of intravenous drug use, of which two patients were not current users. The median Charlson Comorbidity Index Score was 1.0 (IQR 0–6.8). No patients were living with HIV and just under one-third of patients (*n* = 19; 32.8%) had a history of hepatitis C virus (HCV) infection. The median inpatient stay was 15.5 days (IQR 9.3–27.0). There were six (10.3%) self-discharges and two (3.4%) inpatient deaths.

### Clinical management

All patients received antimicrobials prior to dalbavancin, with a median of 3.0 (IQR 2.0–4.0) agents for a median 18.0 DOT (IQR 7.0–28.0). Fifty-seven (98.3%) patients received dalbavancin off licence, often for more than one indication. Forty (69.0%) patients had a captured bacteraemia. Microbiological data are presented in Table 2. The most common diagnoses were ABSSSI, infective endocarditis (IE) and endovascular infection, each diagnosed in 13 (22.4%) patients.

The rationale for using dalbavancin include ‘barriers to conventional therapy’ (*n* = 26; 46.4%), ‘healthcare worker concerns around long-term IV access’ (*n* = 25; 44.6%), ‘patient conduct on the ward’ (*n* = 14; 25.0%) and ‘non-adherence to conventional therapy’ (*n* = 13; 23.2%). These are presented by history of intravenous drug use in Table 2. Two patients had ‘other’ reasons for dalbavancin use; both native valve IE patients, dalbavancin was used due to failed conventional therapies.

Regarding dalbavancin treatment, 55 (94.8%) patients received their first dose as an inpatient. Thirty-five (60.3%) patients had a planned second dose; just over half (*n* = 18 [51.4%]) received this as an outpatient, 10 (28.6) as an inpatient and seven (20%) did not attend. The given doses followed the first by a median of 8.0 (IQR 7.0–8.0) days. Four (6.9%) patients received a third dose and one patient a fourth, all as outpatients a median of 35.5 (IQR 29.5–48.3) and 42.0 days after their first doses A further patient missed a planned fourth dose. The median cumulative dalbavancin dose per patient was 1500 mg (*n* = 56, IQR 1500–3000) given over a median of 1.0 doses (IQR 1.0–2.0). A total of eight (13.8%) patients missed one or more planned dalbavancin dose. Dalbavancin was given in combination with other antimicrobials for 14 patients (24.1%).

### Adverse reactions to dalbavancin

Two patients (3.4%) had an adverse reaction to dalbavancin; one patient experienced phlebitis following their first dalbavancin dose then tolerated their second, another developed drug-induced neutropaenia to a trough of 0.8 × 10^9^/L, which was successfully treated with granulocyte colony-stimulating factor.

### Outcomes

While alive at 90 days, the treatment outcome could not be determined for one patient repatriated to a hospital outside of the Trust. Of the fifty-seven (98.3%) patients with known outcomes, 43 (75.4%) had treatment success. Treatment failed for seven (12.3%) patients, and a further seven were LTF.

Ninety days following their first dalbavancin dose, six patients (10.3%) had died. For four of these patients’ treatment was considered a success and their deaths unrelated to the infection episode. Three patients were PWID whose unexpected community deaths where each determined to be drug-related by the coroner. The fourth patient was not a PWID, and managed with dalbavancin palliatively. Causes of death were unrelated to infection and its treatment. Treatment was considered a ‘failure’ in two cases, neither PWID, both had expected inpatient deaths. One was a 53-year-old with a Charlson Comorbidity Index Score of five treated with dalbavancin empirically for an ABSSSI and IE with palliative rationale after a new diagnosis of metastatic pancreatic cancer. ABSSSI and IE were given as causes of death. The second patient was a 75-year-old with a Charlson Comorbidity Index Score of five treated for a *Streptococcus oralis* bacteraemia and prosthetic valve IE. Dalbavancin was chosen palliatively due to disseminated malignancy. The patient died 18 days after their first and only dose, with cerebrovascular event and IE (treated with 4 weeks of antibiotics) cited as cause.

Data for the seven patients for whom treatment failed are presented in Table 3. Three (42.9%) were PWID. The two cases who died (28.6% of total cases of failure) have already been described. Of patients who failed treatment, fewer had a microbiological diagnosis (three; 42.9% compared with 46; 79.3%) and received previous antibiotics for fewer DOT (9.0 (IQR 5.0–14.0) to 18.0 (IQR 7.0–28.0)). A similar proportion missed a planned dose of dalbavancin. Of the six patients who failed therapy and were discharged, four were readmitted for the same infection episode (4/57; 1.8%).

Of the eight patients who missed a planned dose of dalbavancin, treatment success and failure rates were comparable with those who did not; 75.0% (6/8) versus 75.5% (37/49), and 12.5% (1/8) versus 12.2% (6/49), respectively. The LTF rates were also similar; 12.5% versus 12.2%.

### PWID

Current or recent-past intravenous drug use was reported for 35 patients (60.3%). Comparatively, PWIDs were younger (median age 41.0 versus 72.0 years), and had lower Charlson Comorbidity Index Scores (0 versus 5.0). PWID had high rates of HCV infection (*n* = 19; 54.3% to zero), history of venous thromboembolism (*n* = 18; 51.4% to *n* = 2; 8.7%) and depression (*n* = 9; 25.7% to *n* = 1; 4.3%). Over half (*n* = 18; 56.3%) had insecure housing at the time of admission compared with one (4.3%) in the non-PWID group.

In PWID endovascular infection (31.4% versus 8.7%), native bone and joint infection (20% versus 8.7%) and ABSSSIs (25.7% versus 17.4%) were more common. Microbiology was reflective; MSSA caused a higher proportion of bacteraemias [60.9% (14/23) versus 41.2% (7/18)], *Streptococcus pyogenes* bacteraemia was more common [17.4% (4/23) compared with 0], as were *Streptococcus dysgalactiae* and mixed bacteraemias (both causing 8.7% of bacteraemias compared with zero).

The underlying rationale for choosing dalbavancin differed between PWID and non-PWID. ‘Healthcare worker concerns around long-term intravenous access’ was the commonest reason cited for dalbavancin use in this group (*n* = 21; 60.0%) compared with the non-PWID group (*n* = 4; 19.0%). ‘Patient conduct on ward’ (*n* = 13; 37.1% compared with *n* = 1; 4.8%) and ‘non-adherence to conventional therapy’ (*n* = 11; 31.4% compared with 2; 9.5%) were also more commonly described in the PWID group. Dalbavancin was not used palliatively for any PWID compared with four (19.0%) in the non-PWID group. ‘Antimicrobial contraindications’ informed dalbavancin choice for one PWID (2.9%) compared with five (23.8%) in the non-PWID group.

All eight missed dalbavancin doses (22.9%) were in PWID. The length of admission was shorter for PWID [median 14.0 (IQR 9.5–19.0) days compared with 26.0 days (IQR 10.5–49.5)] and all self-discharges were in this group. The median number of days to discharge following their first dalbavancin dose was 0 (IQR 0–3.0) for PWID compared with 4.0 (IQR 0–11.0) days in the non-PWID group. All seven patients lost to follow-up were in PWID (*n* = 7; 21.2%). Three of the deaths within 90 days of dalbavancin therapy were in PWID. The dalbavancin treatment outcome for all of these patients was a success and their deaths unrelated.

## Discussion

The evidence-base for dalbavancin’s off-licence application is limited, and largely restricted to populations in mainland-Europe or the USA.^7,16–28^ To our knowledge, this is the first published UK data on the use of dalbavancin for off-licence indications, and for PWID outside of the USA.

Nearly all dalbavancin prescriptions in our study were off licence. The most frequent indications were bacteraemia (69.0% of the cohort), ABSSSI, IE and endovascular infection (each diagnosed in 22.4%). With significant heterogeneity, it is difficult to compare studies. In a contemporary 2-year Spanish cohort the most common diagnoses were similar; orthopaedic infections (28.3%), ABSSSIs (22.5%) and cardiovascular infections (20.9%).^16^

Predictably from dalbavancin’s Gram-positive activity, in our study and others, *S. aureus* was the most commonly isolated organism. However, rates of MRSA varied significantly. Of our cohort 8.6% were known MRSA colonized and 8.7 of *S. aureus* bacteraemias were MRSA. This reflects the 7.3% of isolates recorded for the UK by the European Antimicrobial Resistance Surveillance Network (EARS-Net) in 2018.^29^ All studies including PWID have been conducted in the USA, with MRSA rates between 25%^17^ and 88%.^18^ Our study therefore presents useful data on the use of dalbavancin in a low MRSA prevalence context, particularly in PWID.

Unsurprisingly for an agent used off licence, all patients in our cohort received alternative antibiotics before dalbavancin. Previous antibiotic prevalence was high in similar studies; 77.8%^19^ to 100%.^20,30^ Beyond this, the significant variations in treatment reflects the lack of consensus around dalbavancin regimens, especially for off-licence indications, as well as the heterogeneity of studies’ populations and diagnostic indications. Interestingly, a 2020 study where 39.8% of dalbavancin indications were off licence and 62% empirical, found that previous or concomitant antibiotics did not improve the probability of clinical cure using models adjusted for age, comorbidities and site of infection.^19^

Despite being used off licence, dalbavancin caused few (two; 3.4%) adverse reactions, comparable with similar studies.^6,16,20–22,30^ The Morata *et al.* study including significant off-licence prescription did not find any difference in safety at sub-analysis by patient characteristics nor pathogens.^16^ This is encouraging for future use of dalbavancin in a varied population.

Disappointingly a substantial number of our cohort (*n* = 7/58; 12.7%) were LTF. No patients without a history of intravenous drug use were lost to our follow-up in versus 21.2% of PWID, a group for whom this is a recognized challenge.^9^ In comparable studies without PWID, LTF rates range between 3%^21^ and 24.7%.^19^ In reporting studies which include PWID, the proportions LTF are higher, between 15% and 31%.^17,18,23,24^ Heterogeneity in studies’ definitions, populations, representation of PWID, diagnostic indications, follow-up periods and finally the lack of direct comparators, all make efficacy assessments difficult. Few studies have compared dalbavancin with the standard of care for off-licence indications. Of such studies, similar outcomes were seen with dalbavancin when treating osteomyelitis,^25,31^ and the broader indication of invasive Gram-positive infections.^21^ More favourable outcomes were seen in for central line-associated bloodstream infections.^26^ Our outcome data relating to success, failure and LTF are broadly in line with other studies including PWID.^17,18,23,24^ While the small numbers limit useful comparison, it is worth noting that PWID were not over-represented in the seven of our cohort who ‘failed’ treatment. Over 13% of our cohort missed a planned dalbavancin dose. Despite similar treatment outcomes at 90 days, this does raise concerns for drug-resistance in the long-term; an area in need of further study in this context.

### People who inject drugs

PWID represent most of our cohort (60.3%); a population where data is especially sparce. Where included, PWID made up between 30% and 94.4% of study cohorts.^7,17,18,23,24,32–34^ The UK Health Security Agency’s 2018 cross-sectional survey of PWID reported demographics closely reflecting our cohort, suggesting broader generalisability within the UK.^35^ In our study PWID were younger, had fewer comorbidities, but increased rates of depression, HCV and unstable housing: all recognized associations.^10,11^

Diagnoses and organisms treated with dalbavancin in PWID in our study reflect pathogens associated with infections linked to intravenous drug use and the findings of other studies.^7,9–11,24,27,33,34,36^ Although dalbavancin followed comparable therapy, the rationales for its use were different for PWID. For PWID barriers to conventional care often related to intravenous drug use, indeed at the RSCH this history almost excludes outpatient parenteral therapy (OPAT). Although common practice and seen in other studies, evidence suggests that this should not be convention.^9–11,17,23,33^ Studies on OPAT efficacy and safety have found rates of antibiotic completion, line tampering and catheter-associated infections to be comparable in PWID.^10,11^

We note the higher self-discharge rate of 17.1% seen in PWID. This is common in PWID, quoted between 25% and 43%.^10^ Linked with stigma, poor pain and substance-withdrawal management, poor integration with mental health and social services, and prolonged admissions due to no alternatives such as OPAT, unplanned discharge may be reframed as the failure of the health service.^9–11^ This is concerning as unplanned discharges are associated with high rates of readmission, longer subsequent stays and higher 30 day mortality.^10^ Comparative outcome data would be of particular interest in this area.

It is interesting that the time to discharge following the last dose of dalbavancin was shorter in PWID; 0 (IQR 0–3.0) days compared with 4.0 (IQR 0–11.0) days. This hints at the role of dalbavancin in facilitating discharge in a young population with low comorbidity scores. Earlier discharge may also be accommodated by the move towards early switch from intravenous to oral antimicrobials, as can be seen in the OVIVA, POET and SABATO trials. However, evidence about the efficacy of early oral antimicrobial switch in PWIDs remains limited and there are concerns about compliance with oral treatment post-discharge and effective follow-up. Further research is required to compare dalbavancin to early oral antibiotics in this population.^37–39^

When those of our cohort LTF are excluded, treatment success and failure rates for PWID become more comparable to those without this history; success rates of 88.5% (*n* = 23/26) to 82.6% (19/23) and failure rates of 11.5% (*n* = 3/26) to 17.4% (4/23) respectively. Similar to the non-PWID group, the 8.6% 90 day mortality in PWID is strikingly in view of their young ages and low comorbidity scores. A recent matched cohort study of people with a history of illicit opioid use in England found them to have excess risk of death across all major causes of death analysed. In their cohort with a median age of 35.1 years and follow-up period of 8.7 years, 12.4% died.^40^

### Study limitations

Set in a hospital providing District General Hospital as well as tertiary referral hospital services outside of London, these data are probably generalizable to many UK institutions. However, this generalisability is limited by its retrospective and single-centre design. The small participant numbers and currently limited role for dalbavancin will have introduced further bias. We sought to limit selection bias by consecutive recruitment over a 3-year period. The exclusion of outpatient cases and the control of dalbavancin prescription by infection specialists at the RSCH probably biased diagnostic representation. No one in our cohort was living with HIV, and we did not collect data on immunosuppressed states except diabetes. All our patients were treated with antimicrobials before dalbavancin, which must be considered when evaluating efficacy. As there is no standard, various dosing and combination regimens were used for our cohort, limiting the generalisability of efficacy. Whereas we present data on given and missed dalbavancin doses, we did not include planned durations of therapy as this information was usually unavailable. This limits conclusions. The lack of a comparator group makes comparative efficacy assessment impossible. Our study was weakened by the unknown outcome for one patient. The most significant weakness in our study data is the large numbers LTF. Unfortunately, this is common among PWID and highlights the challenges in caring for this population. We did not collect data on the causes of death in our patients which limits inference. Finally, the study period included the years COVID-19 most disrupted hospital and community health care and undoubtedly influenced our findings.

### Conclusions

This cohort offers the first comprehensive review of the real-world experience of dalbavancin use in a UK secondary care setting over a 3-year period. The high proportion of off-licence prescriptions and use in PWID adds to the limited evidence in these areas. Our findings suggest that dalbavancin appears safe and effective when applied to complex infections, particularly when there are barriers to conventional care models. Dalbavancin is a potentially valuable tool in what must be a multifaceted effort to improve the treatment and outcomes for PWID. Further research is required to assess the efficacy, safety, optimal treatment regimens, implications for antimicrobial resistance and healthcare costs of dalbavancin’s real-life use, including in PWID.

## References

[1] Woodford N, Livermore DM. Infections caused by Gram-positive bacteria: a review of the global challenge. J Infect 2009; 59: S4–16. 10.1016/S0163-4453(09)60003-7 19766888

[2] Doernberg SB, Lodise TP, Thaden JT et al Gram-positive bacterial infections: research priorities, accomplishments, and future directions of the antibacterial resistance leadership group. Clin Infect Dis 2017; 64: S24–9. 10.1093/cid/ciw828 28350900 PMC5850444

[3] Bouza E, Finch R. Infections caused by Gram-positive bacteria: situation and challenges of treatment. Clin Microbiol Infect 2001; 7: iii. 10.1046/j.1469-0691.2001.00064.x 11688541

[4] Bennett JE, Dolin R, Blaser MJ. Mandell, Douglas, and Bennett’s Principles and Practice of Infectious Diseases. Elsevier, 2015.

[5] Monteagudo-Martínez N, Solís-García Del Pozo J, Ikuta I et al Systematic review and meta-analysis on the safety of dalbavancin. Expert Opin Drug Saf 2021; 20: 1095–107. 10.1080/14740338.2021.1935864 34042549

[6] Poliseno M, Bavaro DF, Brindicci G et al Dalbavancin efficacy and impact on hospital length-of-stay and treatment costs in different Gram-positive bacterial infections. Clin Drug Investig 2021; 41: 437–48. 10.1007/s40261-021-01028-3 PMC8059686 33884583

[7] Taylor K, Williamson J, Luther V et al Evaluating the use of dalbavancin for off-label indications. Infect Dis Rep 2022; 14: 266–72. 10.3390/idr14020032 35447884 PMC9026399

[8] European Monitoring Centre for Drugs and Drug Addiction (EMCDDA) . European Drug Report: Trends and Developments 2020. Publications Office of the European Union, 2020.

[9] Crepet A, Sargent C. Infections in people who inject drugs on the acute medical take. Clin Med (Lond) 2022; 22: 383–6. 10.7861/clinmed.2022-0364 36507822 PMC9595000

[10] Attwood LO, McKechnie M, Vujovic O et al Review of management priorities for invasive infections in people who inject drugs: highlighting the need for patient-centred multidisciplinary care. Med J Aust 2022; 217: 102–9. 10.5694/mja2.51623 35754144 PMC9539935

[11] Serota DP, Chueng TA, Schechter MC. Applying the infectious diseases literature to people who inject drugs. Infect Dis Clin North Am 2020; 34: 539–58. 10.1016/j.idc.2020.06.010 32782101 PMC8164212

[12] Hickman M, Higgins V, Hope V et al Injecting drug use in Brighton, Liverpool, and London: best estimates of prevalence and coverage of public health indicators. J Epidemiol Community Health 2004; 58: 766–71. 10.1136/jech.2003.015164 15310803 PMC1732885

[13] Charlson ME, Pompei P, Ales KL et al A new method of classifying prognostic comorbidity in longitudinal studies: development and validation. J Chronic Dis 1987; 40: 373–83. 10.1016/0021-9681(87)90171-8 3558716

[14] Stanić Benić M, Milanič R, Monnier AA et al Metrics for quantifying antibiotic use in the hospital setting: results from a systematic review and international multidisciplinary consensus procedure. J Antimicrob Chemother 2018; 73: vi50–8. 10.1093/jac/dky118 29878222 PMC5989607

[15] MHRA . Guidance on Adverse Drug Reactions. 2015: 1–5. https://www.gov.uk/government/uploads/system/uploads/attachment_data/file/403098/Guidance_on_adverse_drug_reactions.pdf.

[16] Morata L, Aguado JM, Salavert M et al Dalbavancin in clinical practice in Spain: a 2 year retrospective study. JAC-Antimicrobial Resist 2022; 4: dlac120. 10.1093/jacamr/dlac120/6957048 PMC9777743 36570687

[17] Morrisette T, Miller MA, Montague BT et al On- and off-label utilization of dalbavancin and oritavancin for Gram-positive infections. J Antimicrob Chemother 2019; 74: 2405–16. 10.1093/jac/dkz162 31322694

[18] Bryson-Cahn C, Beieler AM, Chan JD et al Dalbavancin as secondary therapy for serious *Staphylococcus aureus* infections in a vulnerable patient population. Open Forum Infect Dis 2019; 6: ofz028. 10.1093/ofid/ofz028/5304406 30838225 PMC6388764

[19] Bai F, Aldieri C, Cattelan A et al Efficacy and safety of dalbavancin in the treatment of acute bacterial skin and skin structure infections (ABSSSIs) and other infections in a real-life setting: data from an Italian observational multicentric study (DALBITA study). Expert Rev Anti Infect Ther 2020; 18: 1271–9. 10.1080/14787210.2020.1798227 32797758

[20] Núñez-Núñez M, Casas-Hidalgo I, García-Fumero R et al Dalbavancin is a novel antimicrobial against Gram-positive pathogens: clinical experience beyond labelled indications. Eur J Hosp Pharm 2020; 27: 310–2. 10.1136/ejhpharm-2018-001711 32839266 PMC7447242

[21] Veve MP, Patel N, Smith ZA et al Comparison of dalbavancin to standard-of-care for outpatient treatment of invasive Gram-positive infections. Int J Antimicrob Agents 2020; 56: 106210. 10.1016/j.ijantimicag.2020.106210 33223119

[22] Wunsch S, Krause R, Valentin T et al Multicenter clinical experience of real life dalbavancin use in Gram-positive infections. Int J Infect Dis 2019; 81: 210–4. 10.1016/j.ijid.2019.02.013 30794940

[23] Ajaka L, Heil E, Schmalzle S. Dalbavancin in the treatment of bacteremia and endocarditis in people with barriers to standard care. Antibiotics 2020; 9: 700. 10.3390/antibiotics9100700 33076275 PMC7602462

[24] Vazquez Deida AA, Shihadeh KC, Preslaski CR et al Use of a standardized dalbavancin approach to facilitate earlier hospital discharge for vulnerable patients receiving prolonged inpatient antibiotic therapy. Open Forum Infect Dis 2020; 7: ofaa293. 10.1093/ofid/ofaa293/5870812 32793767 PMC7415304

[25] Cain AR, Bremmer DN, Carr DR et al Effectiveness of dalbavancin compared with standard of care for the treatment of osteomyelitis: a real-world analysis. Open Forum Infect Dis 2022; 9: ofab589. 10.1093/ofid/ofab589/6469809 35071682 PMC8773963

[26] Raad I, Darouiche R, Vazquez J et al Efficacy and safety of weekly dalbavancin therapy for catheter-related bloodstream infection caused by Gram-positive pathogens. Clin Infect Dis 2005; 40: 374–80. 10.1086/427283 15668859

[27] Patel K, Nakagami P, Morita K et al 225. Dalbavancin usage in patients who inject drugs (PWID). Open Forum Infect Dis 2023; 10: ofad500-298. 10.1093/ofid/ofad500.298/7447198

[28] Gonzalez PL, Rappo U, Akinapelli K et al Treatment of acute bacterial skin and skin structure infection with single-dose dalbavancin in persons who inject drugs. Drugs Context 2018; 7: 1–10. 10.7573/dic.212559 PMC6292452 30574170

[29] European Centre for Disease Prevention and Control. *Surveillance of antimicrobial resistance in Europe* 2018. ECDC, 2019. 10.2900/22212.

[30] Arrieta-Loitegui M, Caro-Teller JM, Ortiz-Pérez S et al Effectiveness, safety and cost analysis of dalbavancin in clinical practice. Eur J Hosp Pharm 2022; 29: 55–8. 10.1136/ejhpharm-2020-002315 33020060 PMC8717798

[31] Rappo U, Puttagunta S, Shevchenko V et al Dalbavancin for the treatment of osteomyelitis in adult patients: a randomized clinical trial of efficacy and safety. Open Forum Infect Dis 2019; 6: ofy331. 10.1093/ofid/ofy331/5235615 30648126 PMC6326511

[32] Antosz K, Al-Hasan MN, Lu ZK et al Clinical utility and cost effectiveness of long-acting lipoglycopeptides used in deep-seated infections among patients with social and economic barriers to care. Pharmacy 2021; 10: 1. 10.3390/pharmacy10010001 35076601 PMC8788434

[33] Bork JT, Heil EL, Berry S et al Dalbavancin use in vulnerable patients receiving outpatient parenteral antibiotic therapy for invasive Gram-positive infections. Infect Dis Ther 2019; 8: 171–84. 10.1007/s40121-019-0247-0 31054088 PMC6522607

[34] Bryson-Cahn C, Beieler A, Chan J et al A little bit of dalba goes a long way: dalbavancin use in a vulnerable patient population. Open Forum Infect Dis 2017; 4: S336–7. 10.1093/ofid/ofx163.800 PMC6388764 30838225

[35] UK Health Security Agency (UKHSA) . Unlinked Anonymous Monitoring (UAM) Survey of HIV and Viral Hepatitis Among PWID: 2019 Report, 2019.

[36] Morrisette T, Miller MA, Montague BT et al Long-acting lipoglycopeptides: “Lineless Antibiotics” for serious infections in persons who use drugs. Open Forum Infect Dis 2019; 6: ofz274. 10.1093/ofid/ofz274/5511957 31281868 PMC6602887

[37] Iversen K, Ihlemann N, Gill SU et al Partial oral versus intravenous antibiotic treatment of endocarditis. N Engl J Med 2019; 380: 415–24. 10.1056/NEJMoa1808312 30152252

[38] Li H-K, Rombach I, Zambellas R et al Oral versus intravenous antibiotics for bone and joint infection. N Engl J Med 2019; 380: 425–36. 10.1056/NEJMoa1710926 30699315 PMC6522347

[39] Kaasch AJ, López-Cortés LE, Rodríguez-Baño J et al Efficacy and safety of an early oral switch in low-risk *Staphylococcus aureus* bloodstream infection (SABATO): an international, open-label, parallel-group, randomised, controlled, non-inferiority trial. Lancet Infect Dis 2024: S1473-3099(23)00756-9. 10.1016/S1473-3099(23)00756-9 38244557

[40] Lewer D, Brothers TD, Van Hest N et al Causes of death among people who used illicit opioids in England, 2001–18: a matched cohort study. Lancet Public Heal 2022; 7: e126–35. 10.1016/S2468-2667(21)00254-1 PMC8810398 34906332

